# Protocol for multimodal programming of murine macrophage phenotypes

**DOI:** 10.1016/j.xpro.2026.104767

**Published:** 2026-08-18

**Authors:** Gauri Mirji, Sajad Ahmad Bhat, Rahul S. Shinde

**Affiliations:** 1 Molecular and Cellular Oncogenesis Program, Ellen and Ronald Caplan Cancer Center, The Wistar Institute, Philadelphia, PA 19104, USA; 2 USF Center for Microbiome Research, Microbiomes Institute, Department of Internal Medicine, Morsani College of Medicine, University of South Florida, Tampa, FL 33612, USA

**Keywords:** Cancer, Immunology, Metabolism

## Abstract

Macrophages continuously sense their environment and adopt distinct functional states in response to danger, repair, and homeostatic signals. Here, we present a protocol for generating murine bone marrow-derived macrophages and programming them with microbial/TLR and nucleic acid-sensing agonists, IL-4, apoptotic cells, and tumor microenvironment analogs. We describe steps for stimulation conditions and downstream immunological assays, including flow cytometry, ELISA, RT-PCR, and multiomics, to comprehensively profile murine macrophage phenotypes and functions across these contexts.

For complete details on the use and execution of this protocol, please refer to Mirji et al.^1^; Mirji et al.^2^; and Shinde et al.^3^

## Highlights

• Steps for generating murine bone marrow-derived macrophages from C57BL/6J mice
• Steps for programming macrophages with microbial, IL-4, apoptotic, and tumor cues
• Guidance on profiling macrophage phenotypes by flow cytometry, qPCR, ELISA, and multiomics

## Abstract (editor highlights)

Publisher’s note: Undertaking any experimental protocol requires adherence to local institutional guidelines for laboratory safety and ethics.

## Graphical abstract

**Figure:**
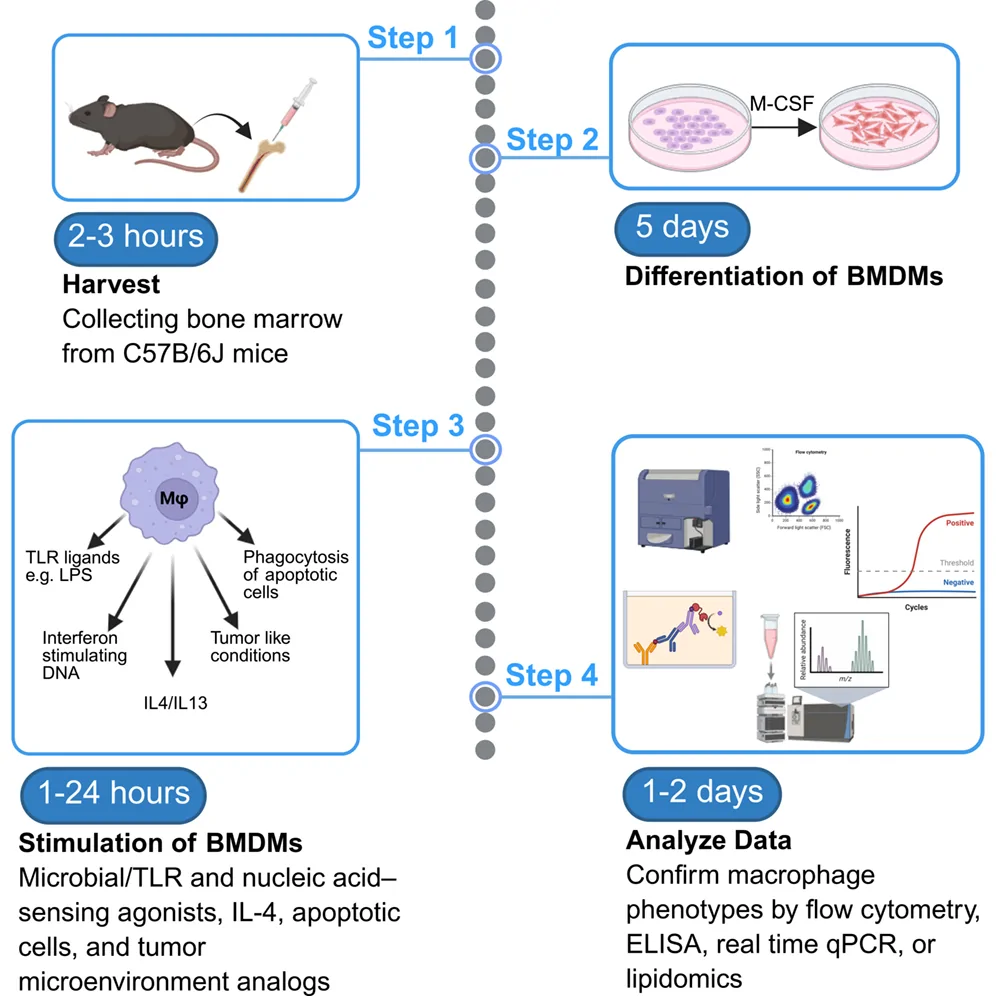

## Before you begin

This protocol describes differentiation of murine BMDMs, stimulating BMDMs with TLR ligands, interferon stimulating DNAs, wound healing stimuli, apoptotic stimuli and tumor like conditioning. Before starting, prepare culture media, buffers, reconstitute and aliquot reagents following the provided recipes.

### Innovation

Macrophages are pivotal regulators of disease pathogenesis and tissue homeostasis. Consequently, accurate modeling of these cells is essential for characterizing their functional responses across diverse pathological states. This protocol describes an expedited and efficient method for the differentiation of murine bone marrow-derived macrophages (BMDMs). While conventional methodologies typically span 6–7 days and require iterative M-CSF restimulation, the current approach achieves a highly pure macrophage population (>90%) within 5 days via a single M-CSF induction. Furthermore, we outline multimodal programming parameters to simulate specific disease-related phenotypes. The utilization of 15 cm^2^ petri dishes enhances macrophage yield, facilitating the execution of multiple downstream functional assays. Ultimately, this protocol provides a robust, scalable, and standardized framework for macrophage research within the fields of immunology, oncology, and infectious disease.

### Institutional permissions

This protocol uses mice, and all animal studies need to be done in accordance with Institutional Animal Care and Use Committee (IACUC) guidelines and must be approved by the IACUC before you begin. Our protocol used 8–12 weeks old female or male mice (C57BL/6J) housed in the animal facility room and fed standard chow.

## Key resources table

**Table:** 

REAGENT or RESOURCE	SOURCE	IDENTIFIER
**Antibodies**		

TruStain FcX PLUS (anti-mouse CD16/32 (Clone S17011E) (diluted 1:200)	BioLegend	Cat# 156604, RRID: AB_2783138
F4/80 PE (Clone BM8) (diluted 1:400)	BioLegend	Cat# 123110, RRID: AB_893486
CD11b BV605 (Clone M1/70) (diluted 1:400)	BioLegend	Cat# 101257, RRID: AB_2565431
MHC-I PerCP/Cyanine5.5 (Clone34-1-2S) (diluted 1:400)	BioLegend	Cat# 114716, RRID: AB_2734176
CD40 PE/Cyanine5 (Clone2/23) (diluted 1:400)	BioLegend	Cat# 124618, RRID: AB_2075922
CD86 BUV395 (Clone GL1) (diluted 1:400)	BD Biosciences	Cat# 564199, RRID: AB_2738664
Arg1 APC (Clone A1exF5) (diluted 1:400)	invitrogen	Cat# 17-3697-82, RRID: AB_2734835
CD206 APC-eFlour 780 (Clone-MR6F3) (diluted 1:400)	invitrogen	Cat# 47-2061-82, RRID: AB_2802285
PDL1 PE/Cyanine7 (Clone 10F.9G2) (diluted 1:400)	BioLegend	Cat# 124314, RRID: AB_10643573

**Chemicals, peptides, and recombinant proteins**		

Lipopolysaccharide (LPS)	Sigma	Cat# L2880
Fixable Viability Dye eFluor 506	Invitrogen	Cat# 65-0866-14
Recombinant mouse MCSF	BioLegend	Cat# 576406
Recombinant IFNγ	BioLegend	Cat# 575306
Recombinant IL-4	BioLegend	Cat# 574304
Lipoteichoic acid	InvivoGen	Cat# tlrl-lta
Pam3CSK4	InvivoGen	Cat# tlrl-pms
Poly (I:C)	InvivoGen	Cat# tlrl-picw
Zymosan	InvivoGen	Cat# tlrl-zyn
R848	InvivoGen	Cat# tlrl-r848
2′3′-cGAMP	InvivoGen	Cat# tlrl-nacga23-02
MDP	InvivoGen	Cat# tlrl-mdp
Ovalbumin (OVA)	Sigma-Aldrich	Cat# A5503
ACK lysis buffer	Quality Biological	Cat# 118-156-101
Powerup SYBR green	Applied Biosystem	Cat# A25742
RLT lysis Buffer	Qiagen	Cat# 74136
ISD (Interferon stimulatory DNA)	InvivoGen	Cat# tlrl-isdn
Poly(dG:dC),	InvivoGen	Cat# tlrl-pgcn
2′3′-cGAMP	InvivoGen	Cat# tlrl-nacga23-02
Lipofectamine 3000 Transfection Reagent	Thermo Fisher Scientific	Cat# L3000001
Opti-MEM™ I Reduced Serum Medium	Thermo Fisher Scientific	Cat# 31985070

**Critical commercial assays**		

RNeasy Plus Mini Kit	Qiagen	Cat# 74136
Annexin V Apoptosis Detection Kit	BioLegend	Cat# 640914
Anti-mouse IL-6 ELISA kit	Invitrogen	Cat# 88-7064
Anti-mouse IL-12p40 ELISA kit	Invitrogen	Cat# 88-7120
Anti-mouse IL-10 ELISA kit	Invitrogen	Cat# 74136
Anti-mouse TGF-β ELISA kit	Invitrogen	Cat# 88-8350-22
Anti-mouse TNF-α ELISA kit	Invitrogen	Cat# 88-7324-22
CellTrace™ Violet Cell Proliferation Kit	Invitrogen	Cat# C34557
iScript™ Reverse Transcription Supermix kit	Bio-Rad	Cat# 1708841

**Oligonucleotides**		

Beta-actin F: 5′- CATTGCTGACAGGATGCAGAAGG-3′ R: 5′- TGCTGGAAGGTGGACAGTGAGG-3′	NA	NA
IRF5 F: 5′- CCTACAGAACCACTCTTGCCTG-3′ R: 5′- CCTTGTGGGTTGCTGATGGTGA-3′	NA	NA
IRF7 F: 5′- CCTCTGCTTTCTAGTGATGCCG-3′ R: 5′- CGTAAACACGGTCTTGCTCCTG-3′	NA	NA
IFNA1 F: 5′- GGATGTGACCTTCCTCAGACTC-3′ R: 5′- ACCTTCTCCTGCGGGAATCCAA-3′	NA	NA

**Experimental models: Organisms/strains**		

C57BL/6J mice (Mus musculus)	Jackson lab	Strain #:000664
Age: 8–10 weeks	NA	RRID:IMSR_JAX:000664
Gender: Male/Female	NA	NA

**Software and algorithms**		

FlowJo v10	BD Bioscience	https://www.flowjo.com/
GraphPad Prism version 10	GraphPad Software	https://www.graphpad.com/
BioRender	BioRender.com	https://biorender.com

## Materials and equipment

**Table:** 

Component	Stock concentration	Solvent/Diluent
LPS	10 mg/mL	Dissolve in sterile DPBS (no calcium, no magnesium) Make 100 μL aliquots and store at −80°C
CpG	5 μM	Dissolve in sterile water. Make 20 μL aliquots and store at −20°C.
Pam3CSK4	1 mg/mL	Dissolve in sterile water. Make 100 μL aliquots and store at −20°C.
Poly I:C	20 mg/mL	Dissolve in sterile water. Make 100 μL aliquots and store at −20°C.
LTA	2.5 mg/mL	Dissolve in sterile water. Make 100 μL aliquots and store at −20°C.
Zymosan	10 mg/mL	Dissolve in sterile water. Make 200 μL aliquots and store at −20°C.
R848	1 mg/mL	Dissolve in sterile water. Make 100 μL aliquots and store at −20°C.
ISD	1 mg/mL	Dissolve in nuclease free water. Make 20 μL aliquots and store at −20°C.
Poly (dG:dC)	1 mg/mL	Dissolve in nuclease free water. Make 20 μL aliquots and store at −20°C.
MDP	5 mg/mL	Dissolve in sterile water. Make 100 μL aliquots and store at −20°C.
2′3′-cGAMP	200 μg/mL	Dissolve in nuclease free water. Make 20 μL aliquots and store at −20°C.

### Complete DMEM

This medium is used to culture and stimulate the murine BMDMs.

**Table:** 

Component	Stock concentration	Final concentration	Volume for 500 mL
DMEM	1×	1×	445 mL
FBS	100%	10%	50 mL
Pen/Strep	100%	1%	5 mL

### Detachment Buffer

This buffer is used to detach the BMDMs from petri dishes.

**Table:** 

Component	Stock concentration	Final concentration	Volume for 500 mL
DPBS (no calcium, no magnesium)	1×	1×	486 mL
EDTA	0.25 M	2 mM	4 mL
FBS	100%	2%	10 mL

Store at 4°C for up to 1 month. Use under sterile conditions.

## Step-by-step method details

### Culturing bone marrow-derived macrophages from C57BL/6J mice

**Timing: 5 days**

In this step, we describe how to start the differentiation of hematopoietic stem cells from bone marrow into mature macrophages.

***Note:*** Ensure all work is performed in a sterile laminar flow hood using aseptic techniques from step 2 onwards. Mice should be euthanized according to your institutional animal care guidelines.

1. Harvest the bone marrow.
  a. Euthanize the mouse and sterilize the hind limbs with 70% ethanol.
  b. Dissect the femurs and tibias, removing as much muscle tissue as possible.

***Note:*** Collect the cleaned bones in complete DMEM kept on ice. Do not expose the bone cavities when dissecting the bones.

**Figure 1:**
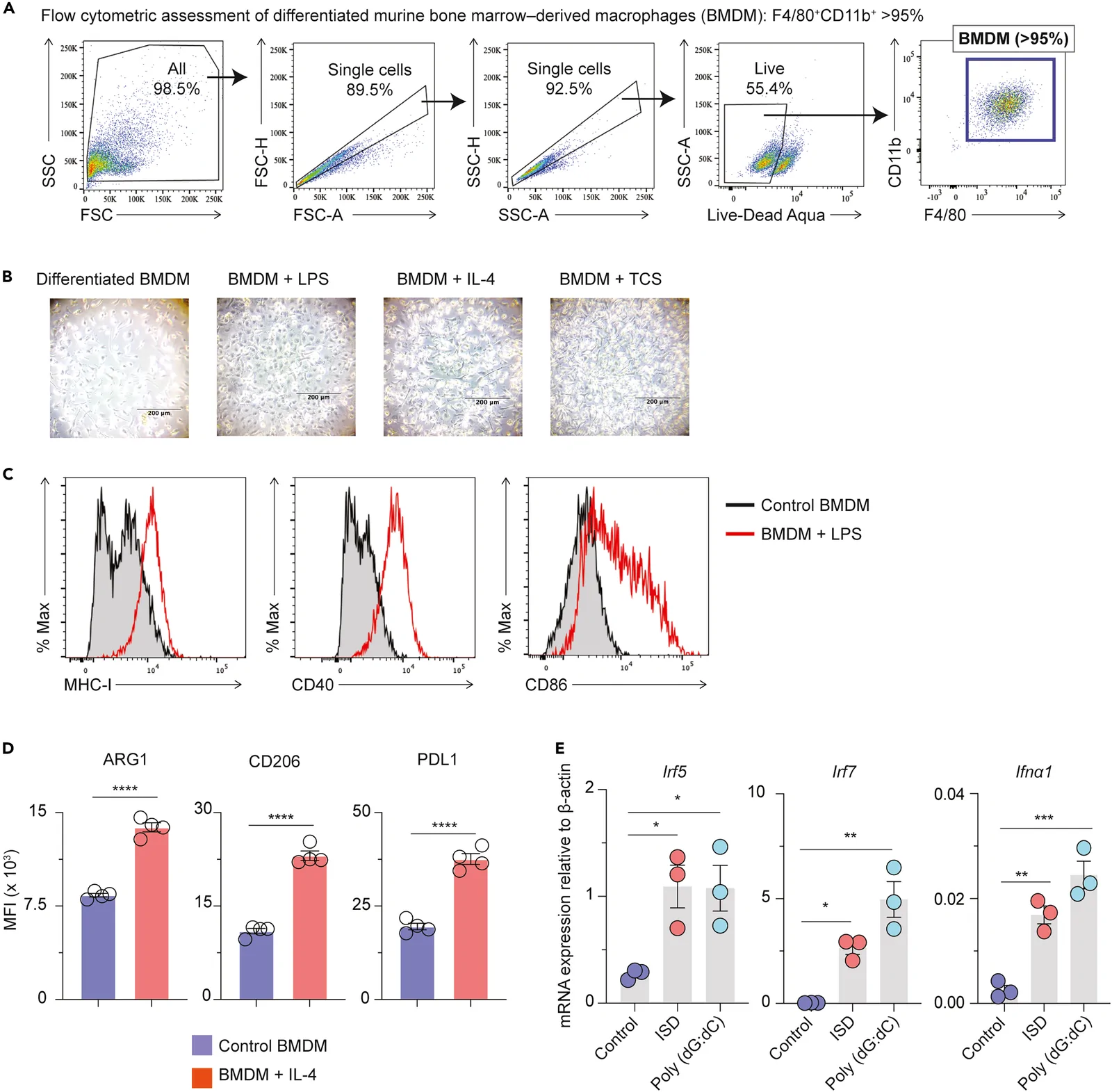
Phenotypes and activation profile of bone marrow derived macrophages (BMDMs) under various inflammatory/suppressive conditions (A) Gating strategy for checking purity of BMDMs on day 5 after differentiation. (B) Morphologies of BMDMs shown with bright field microscopy under untreated, LPS (100 ng/mL) treated, IL-4 (20 ng/mL) treated and TCS (50%) treated conditions. (C) Flow cytometry on BMDMs for activation markers MHC-I, CD40 and CD86 with LPS stimulation (100 ng/mL). (D) Flow cytometry on BMDMs for immunosuppressive markers Arg1, CD206 and PDL1 as MFI. n=4. (E) RT-PCR of interferon stimulated genes *Irf5*, *Irf7* and *Ifnα1* in BMDMs treated with ISD (1 μg/mL) and Poly (dG:dC) (1 μg/mL) for 8 hr. n=3. In (D), P values were determined by two-tailed Student’s t tests. Data are presented as means ± SD and in and (E) P values were determined by one-way ANOVA with post hoc multiple comparisons. Significance is indicated as follows: ∗p < 0.05; ∗∗p < 0.005;∗∗∗p < 0.001; ∗∗∗∗p < 0.0001.

2. Clean the bone marrow.
  a. Cut the ends of the bones to expose the marrow cavity.
  b. Flush the marrow out of the bones into a 10 cm petri dish using a 27-gauge needle attached to a syringe filled with ice-cold, sterile DMEM.
3. RBC lysis.
  a. Centrifuge the cell suspension at 400 × *g* for 5 min at 4°C.
  b. Resuspend the pellet in ACK lysing buffer to lyse the RBCs for 2–5 min at 25°C.
  c. Then add up to 10 mL sterile PBS and centrifuge at 400 × *g* for 5 min at 4°C.
4. Seeding the cells.
  a. Resuspend the pellet in 10 mL complete DMEM.
  b. Filter through 40 μm filter to remove the debris.
    ***Note:*** Filtration step is important after ACK lysis, as the cells tend to clump and dead RBCs and leftover pieces from muscle tissues need to be removed to get a clean single cell suspension.
  c. Then add 20–40 ng/mL M-CSF. Seed 25–35 × 10^6^ cells in 15 cm petri dish with a total 20 mL media per plate.
    **CRITICAL:** M-CSF (Macrophage Colony-Stimulating Factor) is the critical driver for differentiation. Without it, cells will not differentiate into macrophages.
5. Incubate the plates in the incubator at 37°C for 5 days.
6. On Day 5, harvest BMDMs for downstream assays.
  **CRITICAL:** It is not recommended to use trypsin to detach these macrophages from plates.
  ***Note:*** Prepare detachment buffer by adding 10 mL FBS and 4 mL 0.2 M EDTA to 500 mL of cold PBS.
  a. Aspirate the macrophage culture media.
  b. Add 10 mL PBS and aspirate to give a quick wash.
  c. Add 10 mL detachment buffer to each 15 cm^2^ plate.
  d. Incubate the plates at 4°C for 20 min.
  e. Take out the plates from the refrigerator and flush the buffer using the 10 mL pipette.
  f. Collect the cell suspension in a conical tube and centrifuge at 400 × *g* for 5 min at 4°C.
  g. Take a cell count using trypan blue dye and seed the cells in 6 well or 12-well or 24-well plates for experiments at these cell densities:
    i. 24-well: 5.0 × 10^5^ cells/well in 500 μL.
    ii. 12-well: 1 × 10^6^ cells/well in 1 mL.
    iii. 6-well: 2 × 10^6^ cells/well in 2 mL.
      ***Note:*** When seeding 25–35 × 10^6^ cells in a 15 cm Petri dish, the expected recovery is typically ∼8–15 × 10^6^ macrophages, although the final yield may vary depending on the starting material, isolation efficiency, and culture conditions.
      **CRITICAL:** BMDMs are rested for 16–18 h at 37°C incubator in complete DMEM (with 10% FBS, 1% Pen-strep, without M-CSF) before using them for experiments.
      ***Optional:*** Check the purity of differentiated BMDMs using F4/80 and CD11b surface markers and analyze with flow cytometry. The purity of BMDMs should be above 90% as shown in Figure 1A. Check Troubleshooting if the yield is low.

### Stimulation mimicking microbial infection and nucleic acid sensing

**Timing: 1–24 h**

**Timing: 8–24 h (step 7)**

**Timing: 1–24 h (step 9)**

**Timing: 20 min (step 9d)**

In this part, we describe stimulation of murine BMDMs with pathogen-associated molecular patterns (PAMPs) engaging surface and endosomal Toll-like receptors (TLRs), together with cytosolic nucleic acid stimuli that activate the cGAS-STING pathway. Surface TLR ligands (e.g., LPS, LTA, Pam3CSK4, zymosan) primarily model bacterial, fungal, or extracellular microbial sensing, whereas endosomal TLR agonists (R848, CpG) and cytosolic DNA or 2′3′- cGAMP model viral and aberrant self-nucleic acid sensing and promote robust type I interferon responses. This unified section uses a common BMDM preparation and shared readouts (ELISA, RT-qPCR, flow cytometry, multiomics) to enable direct comparison of inflammatory programming across microbial and nucleic acid-sensing contexts.

7. Surface and endosomal TLR ligands mimicking bacterial infection.
  In this step, we describe how to stimulate BMDMs with classic TLR ligands that mimic bacterial and fungal infections such as – Lipopolysaccharide/LPS (TLR4) (Sigma, L2880), Lipoteichoic acid (TLR2) (tlrl-lta; InvivoGen), Pam3CSK4 (TLR2/1) (tlrl-pms; InvivoGen), Zymosan (TLR2) (tlrl-zyn; InvivoGen), R848 (TLR7/8) (tlrl-r848; InvivoGen), CpG (TLR9).
  ***Note:*** Reconstitute all the TLR ligands under sterile conditions based on the manufacturer’s instructions and store at −20°C for up to 4–6 months.
  a. Prepare the TLR ligands in complete DMEM at these concentrations: LPS (100 ng/mL), LTA (1 μg/mL), Pam3CSK4 (100 ng/mL), Zymosan (10 μg/mL), R848 (5 μg/mL), CpG (500 nM).
  b. Aspirate the media from plates and add stimulation medium: 1 mL/well for 12-well plates or 500 μL/well for 24-well plates, based on the plate format used to seed BMDMs.
  c. Incubate at 37°C incubator for 8–24 h.
  d. Harvest the plates for any one of the below readouts:
    i. After 8 h, take out the plates and collect supernatant in a fresh 96-well plate for cytokine measurements by ELISA. Store the 96 well plate with supernatant at −20°C up to 1 to 2 months.
    ii. For RNA isolation, aspirate the media after 8 h. Give a quick wash with PBS. Add 350 μl/well RNA lysis buffer and store the plate at −20°C.
      ***Note:*** Prepare RNA lysis buffer by mixing 350 μL RLT lysis buffer with 20 μL/mL β-mercaptoethanol
    iii. For immune marker analysis, harvest the plate after 24 h and perform flow cytometry (described in a later section). The expected outcome is shown in Figure 1C.
8. NOD agonist muramyl dipeptide (MDP) as a peptidoglycan mimic.
  ***Note:*** Muramyl dipeptide (MDP) is a minimal peptidoglycan motif recognized by NOD2 and can be used to model NOD-dependent responses and TLR–NOD crosstalk in BMDMs.
  a. Prepare MDP (e.g., tlrl-mdp; InvivoGen) stock solution according to manufacturer’s instructions and store at −20°C up to 4–6 months.
  b. Prepare working solutions of MDP in complete DMEM at 1–10 μg/mL as a starting range for BMDMs. Titration may be required to optimize cytokine output.
  c. Stimulate BMDMs in 12- or 24-well plates with MDP alone or in combination with submaximal doses of TLR ligands (e.g., low-dose LPS or Pam3CSK4) to model cooperative NOD–TLR signaling.
  d. Incubate at 37°C for 8–24 h.
  e. Collect supernatants for ELISA (e.g., IL-6, TNF, IL-1β), and/or harvest cells for RNA isolation and qPCR analysis of NOD2-responsive genes and inflammatory transcriptional programs, using the same procedures as for TLR ligands.
9. Cytosolic nucleic acid sensing and STING activation mimicking viral infection.
  This part outlines the stimulation of murine BMDMs with various stimuli such as, interferon-stimulatory DNA (ISD), poly(dG:dC), and 2′3′-cGAMP to model viral infection like innate immune activation and cytosolic DNA sensing via STING. Lipid-based cytosolic delivery is optimized for BMDMs, and downstream immune responses are evaluated by RT-qPCR, Western blotting, and/or ELISA to measure type I interferons and interferon-stimulated genes (ISGs).
  ***Note:*** Reconstitute all reagents under sterile conditions, as described in materials and equipment section, and store at −20°C for up to 4–6 months.
  a. Reconstitute interferon-stimulating ligands in nuclease-free water at these concentrations: ISD (1 mg/mL), poly(dG:dC) (1 mg/mL), 2′3′-cGAMP (200 μg/mL).
  b. Use the interferon-stimulating ligands at these concentrations: ISD (1 μg/mL), poly(dG:dC) (1 μg/mL), 2′3′-cGAMP (5 μg/mL).
    **CRITICAL:** These concentrations provide robust induction of type I IFN in BMDMs; extensive titration is generally not required for initial experiments.
  c. Plate BMDMs one day before stimulation. Incubate for 16–18 h to allow adherence.
    **CRITICAL:** Aim for ∼80%–90% confluence at stimulation. Over-confluent BMDMs can blunt interferon responses.
  d. Prepare ligand–lipid complexes (24-well format; scale as needed).
    Below is a 24-well, per-well setup that is a good starting point for BMDMs.
  e. For each condition, label two sterile tubes per well.
  f. Tube A (ligand in Opti-MEM): Add 50 μL Opti-MEM+ ISD: 0.5 μg per well (→ ∼1 μg/mL final in 500 μL) or poly(dG:dC): 0.5 μg per well or 2′3′-cGAMP: 2.5 μg per well (→ ∼5 μg/mL final).
  g. Tube B (lipid reagent in Opti-MEM): Add 50 μL Opti-MEM+ transfection reagent: 1.5 μL per well (starting point).
  h. Mix Tube A gently, mix Tube B gently.
  i. Combine Tube A + Tube B (final 100 μL complex), gently pipette-mix 3–5 times.
  j. Incubate 15 min at 25°C.
  k. For Mock control: Tube A = Opti-MEM only (no ligand) + Tube B as above.
    ***Note:*** Scaling guide (keep same final concentrations): 12-well: double everything (100 μL + 100 μL; 3 μL reagent; ligand 1 μg ISD/poly(dG:dC); 2 μg cGAMP); 6-Well: 4× everything (200 μL + 200 μL; 6 μL reagent; ligand 2 μg ISD/poly(dG:dC); 4 μg cGAMP).
    **CRITICAL:** If you see cytotoxicity, reduce transfection reagent first (e.g., 1.0 μL in 24-well), then reduce ligand.
  l. Stimulation of BMDMs.
    i. Replace old medium with fresh warm complete DMEM without M-CSF.
    ii. 24-well: 400 μL fresh medium (leave room for complexes).
    iii. Add the 100 μL ligand–lipid complex dropwise to each well to reach 500 μL total.
    iv. Gently rock plate to distribute the reagents to all surface of well.
    v. Incubate at 37°C/5% CO_2_ for 1–24 h depending on the readouts. The expected outcome is shown in Figure 1E.
      ***Optional:*** Inhibitor validation: BMDMs may be pre-treated with the indicated inhibitor for 30–60 min before stimulation, with the inhibitor present for the duration of stimulation.

### Stimulation mimicking wound healing and tissue repair

**Timing: 8–24 h**

This part describes the use of IL-4 (with optional IL-13) to model wound-healing and tissue repair–associated macrophage activation, characterized by induction of genes such as *Arg1*, *Retnla* (*Relmα*), *Chil3* (*Ym1*), *Mrc1* (*CD206*), and metabolic rewiring towards oxidative pathways. These conditions approximate type 2 inflammation and tissue repair programs that are complementary to the infection-like contexts described in Part 2.

10. Prepare BMDMs as described in Part 1 and seed them at the same densities (e.g., 5.0 × 10^5^ cells/well in 24-well plates, 1 × 10^6^ cells/well in 12-well plates).
11. Rest BMDMs for 16–18 h in complete DMEM without M-CSF.
12. Prepare cytokine stimulation medium:
  a. IL-4: 10–20 ng/mL final concentration in complete DMEM without M-CSF.

***Optional:*** IL-13: 10–20 ng/mL, used alone or in combination with IL-4 to further promote alternative activation.

13. Aspirate media and add IL-4–containing medium (±IL-13) to BMDMs (1 mL/well for 12-well plates, 500 μL/well for 24-well plates).
14. Incubate at 37°C for 8–24 h for marker expression and gene expression analysis, or up to 48 h if extended functional assays (e.g., phagocytosis/efferocytosis) are planned.
15. Readouts:
  a. RT-qPCR for *Arg1*, *Retnla*, *Chil3*, *Mrc1*, and genes involved in oxidative metabolism and tissue repair.
  b. Flow cytometry for Arg1, CD206 (MRC1), PD-L1, and other M2-associated markers. The expected outcome is shown in Figure 1D.

***Optional:*** Phagocytosis or efferocytosis assays to assess functional uptake capacity under wound-healing conditions.

### Modeling steady-state homeostasis and tolerance via apoptotic cell clearance

**Timing: 4–24 h**

Apoptotic cell clearance (efferocytosis) is a central mechanism by which macrophages maintain immunological tolerance and prevent systemic autoimmunity. Apoptotic cell–derived signals engage nuclear receptors and transcription factors, including the aryl hydrocarbon receptor (AhR), to induce regulatory and anti-inflammatory programs that suppress pathogenic inflammation, as shown in systemic lupus erythematosus models in mice and humans.^3^

This part describes how to model apoptotic cell–induced tolerogenic programming in BMDMs.

16. Generation of apoptotic cells.
  a. Use primary thymocytes or a suitable lymphoid cell line as a source of apoptotic cells.
  b. Induce apoptosis by:
    i. 40 Gy of radiation in a Gammacell irradiator, followed by 6 h of incubation at 37°C in RPMI 1640 plus 1% BSA.
    ii. Staurosporine (1 μM) treatment and incubation at 37°C in RPMI 1640 plus 1% BSA for 4–6 h.
  c. Wash cells thoroughly with PBS to remove free DNA and soluble factors that may confound readouts.
  d. Confirm apoptosis using Annexin V/propidium iodide staining by flow cytometry, aiming for a high proportion of Annexin V^+^/PI^-^ cells.
17. Co-culture with BMDMs.
  a. Prepare BMDMs as described in Part 1 and seed them at standard densities.
  b. Rest BMDMs for 16–18 h in complete DMEM without M-CSF.
  c. Add apoptotic cells to BMDMs at defined ratios (e.g., 5:1 or 10:1 apoptotic cells to BMDMs) in complete DMEM with 10% FBS and 1% penicillin/streptomycin (Pen/Strep), without M-CSF.
18. Incubate at 37°C:
  a. 2–4 h to assess efferocytosis (uptake of apoptotic cells).
  b. 8–24 h to assess induction of tolerogenic gene expression and cytokine production.
19. Readouts.
  a. Flow cytometry:
    i. Measure uptake of fluorescently labeled apoptotic cells (e.g., CellTrace-labeled apoptotic targets or pH-sensitive dyes) by BMDMs.
    ii. Assess expression of regulatory markers such as Arg1, IL-10, PD-L1, PD-L2, MerTK, and CD36, in comparison with inflammatory markers induced by TLR stimulation in Part 2.
  b. RT-qPCR:
    i. Quantify expression of tolerogenic and regulatory genes, including *Il10*, *Tgfb1*, *Ahr* target genes, *MerTK*, and other genes associated with efferocytic macrophages.
  c. ELISA:
    i. Measure IL-10 and TGF-β secretion as functional outputs of tolerogenic macrophage programming.

***Optional:*** T cell co-culture assay- Co-culture apoptotic cell conditioned BMDMs with antigen-specific T cells to functionally validate their suppressive or tolerogenic capacity. For example, use OVA-expressing apoptotic cells, incubate them with BMDMs to allow efferocytosis, and then add OT-I (CD8^+^, OVA_257–264_–specific) and/or OT-II (CD4^+^, OVA_323–339_–specific) T cells. Measure T cell proliferation (e.g., CFSE or CellTrace dilution) and cytokine production (such as IFN-γ, IL-2, IL-10) as readouts of how apoptotic cell–conditioned macrophages shape antigen-specific effector versus tolerogenic T cell responses.

***Note:*** Combined treatments (e.g., apoptotic cells plus low-dose TLR ligands or microbial metabolites) can be used to dissect how tolerance pathways buffer or modulate inflammatory programming described earlier.

### Stimulation mimicking the tumor microenvironment

**Timing: 8–24 h**

In this step, we describe how to stimulate murine BMDMs with tumor-conditioned media and tumor cell supernatant which mimic the tumor microenvironment.

***Note:*** Prepare tumor conditioned media (TCM) and tumor cell supernatant (TCS) before proceeding with the assay.

20. Preparation of tumor conditioned media (TCM).
  a. Inject C57BL/6J mice with murine cancer cell lines (e.g., 2838c3 murine PDAC) either by subcutaneous or orthotopic injections.
  b. Harvest the mice after 21 days or when tumor volume is 500–700 mm^3^.
  c. Collect the tumors in complete DMEM containing petri dish.
  d. Weigh the tumors.
    ***Note:*** Perform the following steps aseptically inside a laminar hood.
  e. For every 200 mg tumor tissue add 1 mL complete DMEM. For e.g., if the total tumor weight is 5 g, then take 25 mL complete DMEM in 15 cm^2^ petri dish.
  f. Cut the tumors into small pieces and then crush the tumor pieces with the end of syringe plunger.
  g. Incubate the tissue suspension for 2 hr at 37°C.
  h. Centrifuge tissue suspension at 400 × *g* for 5 min.
  i. Collect the supernatant in 1.5 mL centrifuge tubes and centrifuge 21,000 × *g* for 10 min to remove cellular/tissue debris.
  j. Collect the supernatant and filter using sterile 0.2 μm filters (VWR, 28145-477).
  k. The filtrate is TCM, it can be stored at −20°C for up to 4–6 months.
21. Stimulate BMDMs with TCM.
  a. Continuing from step 6-g, Aspirate the media from 12 or 24 well plates.
  b. Add TCM to each well with final concentration of 20%.
  c. Incubate the plates at 37°C for 8 h for RNA isolation or 24 h for flow cytometry readouts.
22. Preparation of Tumor Cell Supernatant (TCS).
  a. Culture the murine cancer cell lines (e.g., 2838c3 murine PDAC) in complete DMEM media.
  b. Sub-culture/split the cells when the flask is 70%–80% confluent.
    ***Note:*** Keep the spent media aside before trypsinization.
  c. Detach the cells with trypsin-EDTA, spin at 400 × *g* for 5 min.
  d. Resuspend the pellet in complete DMEM.
  e. Seed the cells in fresh flask with half fresh and half spent media (e.g., 6 mL fresh media + 6 mL spent media).
  f. Let the cells become 80%–90% confluent.
  g. Collect the spent culture media.
  h. Spin at 400 × *g* for 5 min.
  i. Collect the supernatant and freeze at −20°C for up to 4–6 months.
    ***Optional:*** Filter the supernatant through 0.2 μm filter to get clear cell free TCS.
23. Stimulate BMDMs with TCS.
  a. Continuing from step 6-g, Aspirate the media from 12 or 24 well plates.
  b. Add TCS to each well with final concentration of 50%.
  c. Incubate the plates at 37°C for 8 h for RNA isolation or 24 h for flow cytometry readouts.

## Expected outcomes

BMDMs stimulated under the influence of different inflammatory conditions (microbial/TLR and nucleic acid sensing, wound-healing cytokines, apoptotic cells, and tumor microenvironment) secrete variety of cytokines and express multiple markers and intracellular markers, as well as distinct transcriptional programs.

Flow cytometry serves as a primary tool for characterizing these cells, involving a detailed process of harvesting cells via gentle scraping and staining for both surface and intracellular markers. To capture the full phenotypic spectrum, use specific antibody cocktails and stimulation techniques-such as PMA and ionomycin, to detect activation and intracellular markers like CD86, CD206 and PD-L1 and Arg1 respectively. As shown in Figure 1C, the activation markers can be quantified using % positive expression or as mean fluorescence intensity (MFI) as shown in Figure 1D in stimulated conditions compared to control murine BMDMs. Data acquisition is typically performed on high-end platforms like the BD FACS Symphony, with subsequent analysis with FlowJo to interpret the complex marker profiles.^1^^,^^2^

Complementing the protein-level data, gene expression and cytokine secretions are quantified through real-time qPCR and ELISA respectively. As shown in Figure 1E, the relative expression of interferon stimulated genes can be quantified in murine BMDMs stimulated with ligands mimicking viral infection. For quantification of secreted cytokines like TNF, IL-6, and TGF-β, ELISA can be performed on culture supernatants from these different conditions. Macrophage lipidome can be analyzed after inflammatory or tumor-like stimulations with sophisticated lipidomics.^1^^,^^4^ This process involves extracting lipids using ice-cold methanol and a chloroform-based solvent system, spiked with stable isotope-labeled standards for accuracy. The resulting samples are analyzed via LC-MS/MS high-resolution mass spectrometry to identify specific lipid species, providing a high-definition view of the macrophage metabolic state under different pathological stimuli.

## Limitations

This protocol is based on murine BMDMs cultured *in vitro*, providing a controlled and reproducible system but not fully capturing the diversity of tissue-resident or human macrophages. Responses to the stimuli described here may therefore differ across species, tissues, sex, and disease settings. The microbial/TLR, nucleic acid–sensing, IL-4, apoptotic cell, and tumor-conditioned stimuli represent major axes of macrophage programming but do not encompass the full range of signals present in intact tissues over time. Ligand concentrations and time points are optimized for robust bulk responses and may miss subtle dose–response effects or single-cell heterogeneity without additional titration and kinetic studies. Finally, tumor-conditioned media and tumor cell supernatant are practical surrogates for the tumor microenvironment but do not reproduce tissue architecture, oxygen and nutrient gradients, or chronic metabolic stress in solid tumors.

## Troubleshooting

### Problem 1

Suboptimal BMDM yield, poor differentiation, or reduced viability.

### Potential solution

• Check that bone marrow is flushed thoroughly from femurs and tibias and that residual muscle and bone fragments are removed before culture.
• Verify that M-CSF is active and used at the recommended final concentration (20–40 ng/mL) and avoid extending differentiation much beyond 5–6 days.
• Use the EDTA/FBS-based detachment buffer rather than trypsin, and handle cells gently during harvesting and reseeding to preserve viability.

### Problem 2

Weak cytokine or gene induction after TLR, MDP, or STING stimulation.

### Potential solution

• Prepare fresh working solutions from correctly stored ligand stocks and double-check the final concentrations for each stimulus.
• Stimulate BMDMs at roughly 80%–90% confluence and include a robust positive control such as LPS in every experiment to confirm responsiveness.
• For STING agonists, confirm that nucleic acid–lipid complexes are assembled as described and, if toxicity is an issue, lower the amount of transfection reagent before adjusting ligand dose.

### Problem 3

Limited induction of IL-4–responsive wound-healing markers.

### Potential solution

• Confirm the activity and final concentration of IL-4 (with or without IL-13, typically 10–20 ng/mL) and consider extending stimulation to 24–48 h.
• Rest BMDMs for 16-18 h in complete DMEM without M-CSF before adding IL-4 and avoid unintentional exposure to strong inflammatory stimuli unless that is part of the experimental design.

### Problem 4

Poor apoptotic cell uptake or lack of tolerogenic readouts.

### Potential solution

• Optimize the induction of apoptosis (for example, by adjusting UV dose or dexamethasone treatment) and confirm an Annexin V^+^/PI^-^–enriched population with minimal necrosis.
• Wash apoptotic cells thoroughly, use defined target-to-macrophage ratios (such as 5–10:1), and time collection points to capture both efferocytosis (2–4 h) and downstream IL-10/TGF-β production and gene expression changes (8–24 h).

### Problem 5

Variable or unexpected responses to tumor conditioned media or tumor cell supernatant.

### Potential solution

• Standardize tumor models as much as possible, including cell line passage, inoculated cell number, tumor size at harvest, and timing of TCM/TCS collection.
• Process TCM/TCS consistently (clarification, optional filtration, and storage conditions), use fixed percentages in culture, and include internal reference batches or control media to help interpret variability between experiments.

## Resource availability

### Lead contact

Further information and requests for resources and reagents should be directed to and will be fulfilled by the lead contact, Rahul S. Shinde (rsshinde@usf.edu).

### Technical contact

Technical questions on executing this protocol should be directed to and will be answered by the technical contacts, Gauri Mirji (gmirji@wistar.org) and Sajad Ahmad Bhat (sbhat@wistar.org).

### Materials availability

This study did not generate new unique reagents. All materials are purchasable.

### Data and code availability

This study did not generate code.

## Acknowledgments

This work was supported by grants from the 10.13039/100000002National Institutes of Health (NIH; R37 CA280869, R01 CA293027, and R21 CA259240) and by a grant from the Margaret Q. Landenberger Research Foundation. We thank the Animal Facility, Flow Cytometry Facility, Genomics Facility, Bioinformatics Facility, and Proteomics & Metabolomics Facility at The Wistar Institute for their technical support and services.

## Author contributions

R.S.S.: supervision, funding acquisition, conceptualization, investigation, data collection, methodology, writing – original draft, and writing – review and editing; G.M.: investigation, data collection, methodology, formal analysis, investigation, writing – original draft, writing – review and editing, and visualization; S.A.B.: investigation, data collection, methodology, formal analysis, investigation, writing – review and editing, and visualization.

## Declaration of interests

The authors declare no competing interests.

## Declaration of generative AI and AI-assisted technologies in the writing process

During the preparation of this work, the authors used OpenAI in order to improve writing, grammar, and readability. After using this tool/service, the authors reviewed and edited the content as needed and take full responsibility for the content of the published article.
